# Protein–Sol: a web tool for predicting protein solubility from sequence

**DOI:** 10.1093/bioinformatics/btx345

**Published:** 2017-05-29

**Authors:** Max Hebditch, M Alejandro Carballo-Amador, Spyros Charonis, Robin Curtis, Jim Warwicker

**Affiliations:** 1 School of Chemical Engineering and Analytical Science, Manchester Institute of Biotechnology, University of Manchester, Manchester, UK; 2 School of Chemistry, Manchester Institute of Biotechnology, University of Manchester, Manchester, UK

## Abstract

**Motivation:**

Protein solubility is an important property in industrial and therapeutic applications. Prediction is a challenge, despite a growing understanding of the relevant physicochemical properties.

**Results:**

Protein–Sol is a web server for predicting protein solubility. Using available data for *Escherichia coli* protein solubility in a cell-free expression system, 35 sequence-based properties are calculated. Feature weights are determined from separation of low and high solubility subsets. The model returns a predicted solubility and an indication of the features which deviate most from average values. Two other properties are profiled in windowed calculation along the sequence: fold propensity, and net segment charge. The utility of these additional features is demonstrated with the example of thioredoxin.

**Availability and implementation:**

The Protein–Sol webserver is available at http://protein-sol.manchester.ac.uk.

## 1 Introduction

Protein solubility is an important property, from recombinant protein production to the development of biotherapeutics. A number of methods have been used to predict aggregation (Agrawal *et al.*, 2011) and solubility, based on factors such as propensity to form inclusion bodies (Wilkinson and Harrison, 1991) and β-strands (Tartaglia and Vendruscolo, 2008), structural genomics studies (Magnan *et al.*, 2009), and physicochemical properties (Agostini *et al.*, 2014). A web server is presented, Protein–Sol, for predicting protein solubility, based on the observation of a bimodal distribution of protein solubilities for *E.coli* proteins in cell-free expression (Niwa *et al.*, 2009). These measurements report the amount of a protein that is soluble (in the supernatant subsequent to centrifugation) compared with the total amount of that protein, rather than a thermodynamic property. A wider significance is apparent from two factors. First, that proteins tend to evolve to a point at which their solubility matches that required for their natural abundance (Tartaglia *et al.*, 2007). Second, the properties seen in the current work that associate with more soluble proteins are those seen previously, such as fewer amino acids with aromatic sidechains, favouring negative charge, and a preference for lysine over arginine (Warwicker *et al.*, 2014).

## 2 The Protein–Sol server

Protein–Sol is available at http://protein-sol.manchester.ac.uk without account registration or licence. It processes amino acid sequence and calculates predicted solubility and other properties, which returned in a graphical format and as a text file. Thirty-five features are considered in the algorithm, 20 amino acid compositions; 7 composites: K-R, D-E, K+R, D+E, K+R-D-E, K+R+D+E, F+W+Y; and 8 further predicted features: length, pI, hydropathy (Kyte and Doolittle, 1982), absolute charge at pH 7, fold propensity (Uversky *et al.*, 2000), disorder (Linding *et al.*, 2003), sequence entropy, and β-strand propensity (Costantini *et al.*, 2006). A linear model combining the 35 features gave an initial fit to the solubility data (Niwa *et al.*, 2009). Weights were then derived from differences between the lower and higher 5% tails of the solubility distribution, recorded as z-scores. Proteins predicted to have a transmembrane (TM) segment (hydropathy > 1.6 in any 21 amino acid segment), were excluded.

For a query sequence, the contribution of each feature to predicted solubility is a linear scaling between its corresponding values averaged within each of the lower and higher subsets, multiplied by feature weight, with feature weights normalized to sum to 1. As there are many correlations between features, and because some features do not contribute to the prediction, overall correlation of prediction to the population of experimental solubilities for 2395 proteins, (without predicted TM regions), was used to assess combinations of features, eliminating first those with least weighting, continuing elimination until the model performance falls. The final prediction scheme consists of 10 features (H, L, V, K-R, D+E, F+W+Y, length, absolute charge, fold propensity, sequence entropy), with a correlation coefficient of 0.621 between calculated and experimental values, and 58% predicted solubility giving the best separation threshold of lowest and highest 5% subsets in a receiver operating characteristic (ROC) analysis. In addition to charge-based features, non-polar features are also present in the model. For example, aromatic (F+W+Y) composition weights predicted solubility down, whilst valine weights solubility up. In addition, predicted fold propensity and sequence entropy have a negative influence on predicted solubility. Our interpretation is that, in addition to a charged protein surface being favourable for solubility, there may also be a subset of more soluble proteins that have reduced sequence complexity, perhaps similar to intrinsically disordered proteins. Display of the extent to which each feature deviates from population average allows the user to select features that could be targeted to improve solubility. Net charge and fold propensity over a sliding window are displayed as profiles, providing additional information with which to interpret protein behaviour.

Prediction of solubility from sequence is a single step process for the user. Each sequence for calculation is assigned a unique id number, formatted, and stored temporarily on the server. No calculation occurs if the input is invalid and the user is informed of the mismatch. The algorithm generates a text file that is processed using shell scripts and R to produce a graphical interpretation of the results. The predicted protein solubility is not valid for membrane proteins, but the results will be presented, with a warning, if a predicted transmembrane region is identified.

Several tests have been made of the server. Protein expression data from structural genomics projects is often aggregated and heterogeneous. The first test set consists of 679 strongly expressed and well-behaved proteins from a single pipeline, which were used to derive a model for crystallization propensity (Price *et al.*, 2009). We predict an average solubility of 70.6% for these 679 proteins, with 70.3% of the set above the 58% threshold. A further set of 200 proteins used to test the crystallization model (Price *et al.*, 2009) gives an average of 76.1% predicted solubility with 82.5% of the set above the 58% threshold. Thermophile proteins have evolved to counter particularly stringent tests on solubility (Greaves and Warwicker, 2007). *Methanopyrus kandleri* is a sequenced archaeon with one of the highest known growth temperatures (80−110°C, Slesarev *et al.* 2002). Excluding those containing a predicted TM segment, solubility predictions for 1294 proteins from UniProt (UniProt Consortium, 2017) averaged 78.6%, with 93.6% of these above 58%.

**Fig. 1:**
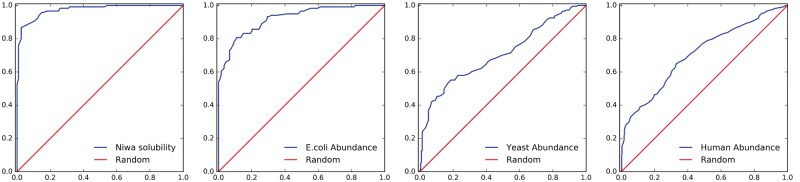
Performance of the predictions across bacterial and eukaryotic proteomes. ROC plots are shown for prediction performance in the training set of measured solubilities, and 3 test sets of protein abundance

A link between protein aggregation rates and gene expression levels (Tartaglia *et al.*, 2007) has been reinforced with comparison of the abundant proteins serum albumin and myoglobin with their less abundant paralogues (Warwicker *et al.*, 2014). Quantitative proteomics allows comparison of (log scale) protein abundance and predicted protein solubility, with ROC plot analysis using low and high abundance subsets from the 5% tails. Calculations have been made with whole proteome integrated sets for *Escherichia coli*, *Saccharomyces cerevisiae* and *Homo sapiens* retrieved from PaxDb (Wang *et al.*, 2015). Results are reported in Table 1 (excluding proteins containing predicted TM segments), with the original development set of *E.coli* protein solubility added for reference. With membrane proteins included (not shown), the measures of agreement increase, an outcome of the importance of charge for protein solubility. Accuracy for the ROC analysis is listed at 58% solubility prediction, since this gives the highest accuracy for the development set. ROC plots are shown in Figure 1.

**Table 1.** ROC plot and correlation analysis of predictions versus protein solubility or abundance

Set	Proteins	5% Tails	AUC	Acc at 58%	Corr
*E.coli* solubility Train	2395	120	0.974	0.900	0.621
*E.coli* abundance Test	2364	119	0.922	0.828	0.382
Yeast abundance Test	4275	214	0.707	0.626	0.188
Human abundance Test	10662	534	0.708	0.659	0.190

*Note*: AUC is area under the curve, Acc is accuracy at 58% solubility prediction threshold.

Through these varied tests, a structural genomics pipeline, the proteome of a hyperthermophile, and protein abundance in organisms across the tree of life, the model consistently demonstrates correlations.

## 3 Discussion

**Fig. 2:**
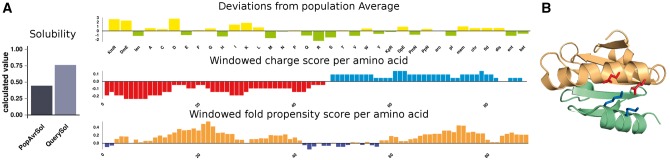
(**A**) The Protein–Sol calculation. Results are shown for the *E.coli* thioredoxin example. (**B**) *E.coli* thioredoxin (2trx chain A, Katti *et al.* (1990)) is shown colour-coded by subdomain (1–67 and 68–108), with salt-bridges E44-K96 and E48-K100 displayed between the subdomains. Drawn with PyMOL (http://pymol.org)

Protein–Sol is demonstrated with *E.coli* thioredoxin, known to enhance solubility of co-produced proteins in *E.coli* (Yasukawa *et al.*, 1995). Predicted solubility (scaled from 0 to 1) is plotted (Fig. 2A) alongside the population average for the experimental dataset (Niwa *et al.*, 2009). Thioredoxin at 0.76 is well above the average of 0.45, consistent with its wider use in co-expression or as a fusion partner. Solubility prediction on the server is given in the 0–1 range for ease of user interpretation. Percentage values, which were used in training and testing, can exceed 100% in the experimental dataset. For reference, thioredoxin predicts at 88% against a population average of 53%. The predicted pI is also displayed. Next, a plot shows deviations from population averages for the 35 features. Although only 10 of these contribute to the prediction, the signed deviations show the characteristics of the input sequence. For example KmR, meaning K-R, is prominent for thioredoxin and contributes to a prediction of highly soluble. To improve solubility, K-R is perhaps more useful than the other 9 features in the final model, since lysine and arginine can generally be swapped with little consequence for protein function or fold. The plot of windowed fold propensity (Fig. 2A) shows two subdomains, consistent with experimental characterization of thioredoxin folding (Katti *et al.*, 1990). The subdomain structure is also apparent in a novel representation of windowed net charge with negatively charged N-terminal and positively charged C-terminal subdomains (Fig. 2B). Whilst the windowed net charge does not indicate a complete separation of charge between subdomains, it shows the possibility for interactions dependent on the opposite sign of net charges, exemplified by the two salt-bridges shown in Figure 2B.

Protein–Sol provides a fast sequence-based method for predicting protein solubility and lysine and arginine content are highlighted in regard to modifying protein solubility, as K/R swaps are likely to be structurally and functionally neutral. A case study with thioredoxin shows that additional features of the server can be used to interpret subdomain structures and introduces the novel feature of windowed net charge, which may inform on charge-charge interactions between subdomains.

## References

[btx345-B1] Agostini F. et al (2014) ccSOL omics: a webserver for large-scale prediction of endogenous and heterologous solubility in E. coli. Bioinformatics, 30, 2975–2977. 24990610 10.1093/bioinformatics/btu420 PMC4184263

[btx345-B2] Agrawal N.J. et al (2011) Aggregation in protein-based biotherapeutics: computational studies and tools to identify aggregation-prone regions. J. Pharm. Sci., 100, 5081–5095. 21789769 10.1002/jps.22705

[btx345-B3] Costantini S. et al (2006) Amino acid propensities for secondary structures are influenced by the protein structural class. Biochem. Bioph. Res. Commun., 342, 441–451. 10.1016/j.bbrc.2006.01.159 16487481

[btx345-B4] Greaves R.B., Warwicker J. (2007) Mechanisms for stabilisation and the maintenance of solubility in proteins from thermophiles. BMC Struct. Biol., 7, 18. 17394655 10.1186/1472-6807-7-18 PMC1851960

[btx345-B5] Katti S. et al (1990) Crystal structure of thioredoxin from *Escherichia coli* at 1.68 Å resolution. J. Mol. Biol., 212, 167–184. 2181145 10.1016/0022-2836(90)90313-B

[btx345-B6] Kyte J., Doolittle R.F. (1982) A simple method for displaying the hydropathic character of a protein. J. Mol. Biol., 157, 105–132. 7108955 10.1016/0022-2836(82)90515-0

[btx345-B7] Linding R. et al (2003) GlobPlot: exploring protein sequences for globularity and disorder. Nucleic Acids Res., 31, 3701–3708. 12824398 10.1093/nar/gkg519 PMC169197

[btx345-B8] Magnan C.N. et al (2009) SOLpro: accurate sequence-based prediction of protein solubility. Bioinformatics, 25, 2200–2207. 19549632 10.1093/bioinformatics/btp386

[btx345-B9] Price W.N. II et al (2009) Understanding the physical properties controlling protein crystallization. Nature Biotechnol., 27, 51–57. 19079241 10.1038/nbt.1514 PMC2746436

[btx345-B10] Niwa T. et al (2009) Bimodal protein solubility distribution revealed by an aggregation analysis of the entire ensemble of Escherichia coli proteins. Proc. Natl. Acad. Sci. USA, 106, 4201–4206. 19251648 10.1073/pnas.0811922106 PMC2657415

[btx345-B11] Slesarev A.I. et al (2002) The complete genome of hyperthermophile Methanopyrus kandleri AV19 and monophyly of archaeal methanogens. Proc. Natl. Acad. Sci. USA, 99, 4644–4649. 11930014 10.1073/pnas.032671499 PMC123701

[btx345-B12] Tartaglia G.G., Vendruscolo M. (2008) The Zyggregator method for predicting protein aggregation propensities. Chem. Soc. Rev., 37, 1395–1401. 18568165 10.1039/b706784b

[btx345-B13] Tartaglia G.G. et al (2007) Life on the edge: a link between gene expression levels and aggregation rates of human proteins. Trends Biochem. Sci., 32, 204–206. 17419062 10.1016/j.tibs.2007.03.005

[btx345-B14] UniProt Consortium (2017) UniProt: the universal protein knowledgebase. Nucleic Acids Res., 45, D158. 27899622 10.1093/nar/gkw1099 PMC5210571

[btx345-B15] Uversky V.N. et al (2000) Why are “natively unfolded” proteins unstructured under physiologic conditions? Proteins, 41, 415–427. 11025552 10.1002/1097-0134(20001115)41:3<415::aid-prot130>3.0.co;2-7

[btx345-B16] Wang M. et al (2015) Version 4.0 of PaxDb: protein abundance data, integrated across model organisms, tissues, and cell-lines. Proteomics, 15, 3163–3168. 25656970 10.1002/pmic.201400441 PMC6680238

[btx345-B17] Warwicker J. et al (2014) Lysine and arginine content of proteins: computational analysis suggests a new tool for solubility design. Mol. Pharm., 11, 294–303. 24283752 10.1021/mp4004749 PMC3885198

[btx345-B18] Wilkinson D.L., Harrison R.G. (1991) Predicting the solubility of recombinant proteins in *Escherichia coli*. Bio/Technology, 9, 443–448. 1367308 10.1038/nbt0591-443

[btx345-B19] Yasukawa T. et al (1995) Increase of solubility of foreign proteins in *Escherichia coli* by coproduction of the bacterial thioredoxin. Increase of solubility of foreign proteins in *Escherichia coli* by coproduction of the bacterial thioredoxin. J. Biol. Chem., 270, 25328–25331. 7592692 10.1074/jbc.270.43.25328

